# Phytotoxicity Evaluation of Type B Trichothecenes Using a *Chlamydomonas reinhardtii* Model System

**DOI:** 10.3390/toxins6020453

**Published:** 2014-01-28

**Authors:** Tadahiro Suzuki, Yumiko Iwahashi

**Affiliations:** Applied Microbiology Division, National Food Research Institute, 2-1-12 Kannon-dai, Tsukuba, Ibaraki 305-8642, Japan; E-Mail: suzut@affrc.go.jp

**Keywords:** type B trichothecene, *Chlamydomonas reinhardtii*, PPFD, LED, toxicity testing

## Abstract

Type B trichothecenes, which consist of deoxynivalenol (DON) and nivalenol (NIV) as the major end products, are produced by phytotoxic fungi, such as the *Fusarium* species, and pollute arable fields across the world. The DON toxicity has been investigated using various types of cell systems or animal bioassays. The evaluation of NIV toxicity, however, has been relatively restricted because of its lower level compared with DON. In this study, the *Chlamydomonas reinhardtii* testing system, which has been reported to have adequate NIV sensitivity, was reinvestigated under different mycotoxin concentrations and light conditions. The best concentration of DON and NIV, and their derivatives, for test conditions was found to be 25 ppm (2.5 × 10^−2^ mg/mL). In all light test conditions, DON, NIV, and fusarenon-X (FusX) indicated significant growth inhibition regardless of whether a light source existed, or under differential wavelength conditions. FusX growth was also influenced by changes in photon flux density. These results suggest that *C. reinhardtii* is an appropriate evaluation system for type B trichothecenes.

## 1. Introduction

Trichothecene mycotoxins are produced by phytotoxic fungi such as the *Fusarium* species, which cause Fusarium head blight. These toxic fungi cause disease damage not only to plants that are used for food and feed crops but also to livestock and humans [1,2]. Trichothecene mycotoxins are categorized into several types and the type B trichothecene group includes deoxynivaelnol (DON), nivalenol (NIV), and their derivatives. They have a relatively low toxicity compared with type A trichothecenes, such as T-2 or HT-2 toxins, but the toxicity varies with differences in cell type or species. The general toxicity mechanism of trichothecene is 60S ribosome binding, leading to translation inhibition [3]. This also causes inhibition of cellular regeneration and, consequently, trichothecene exposure can cause abdominalgia or diarrhea derived from cell inflammation. Because trichothecene mycotoxins pollute food crops and feed grains all over the world, several countries have been establishing restriction values against DON [4]. The restriction value is based on the results of evaluation studies. However, a comprehension of toxic characters is not always sufficient because of the differences in toxic characters between cell types or species. Recently, a number of researchers have suggested that a difference in import ability of the cellular transporter makes the difference in toxicity [5,6,7,8]. A *Saccharomyces cerevisiae* deletion mutant of the *PDR5* gene, which encodes a pleiotropic drug resistance ABC transporter located on the plasma membrane, has increased DON sensitivity [8,9]. However, the deletion of the *PDR5* gene does not result in high sensitivity to NIV [9], but Fusaronon-X (FusX, 4-acetyl-nivalenol), which is a NIV derivative, indicates a high toxicity [10]. Yeast evaluation systems cannot report NIV toxicity well. It is thought that NIV is not retained in yeast cells because of its structure. However, NIV indicates a relatively high toxicity to mammalian cells where it depends on the different cell types [11,12]. Mycotoxins also indicate toxicities to various plants. Abbas *et al*. [12] also reported the phytotoxicity of type B trichothecenes against *Lemna pausicostata* (duckweed), and the result of this study provides distinct phytotoxicity data. It has been indicated that duckweed is useful for toxicity testing [13], and recently, there are indications that duckweed research is developing. However, there is not yet enough genomic information, which is essential for understanding the mechanism of phytotoxicity. Meanwhile, former studies with barley leaf, *Arabidopsis thaliana*, or wheat roots have indicated a high toxicity of DON [14,15,16]. Toxicity evaluation with *Arabidopsis* seems to be a useful test system for trichothecene mycotoxins because several studies have suggested DON or T-2 toxin toxicities [17,18,19]. As for the study with wheat tissue segments, detailed phenotypic toxicity reactions to the mold invasion or the *fusarium* mycotoxin have been reported [20]. However, information about NIV toxicity is not enough even with plant test systems as plant systems have several problems. For example, the *Arabidopsis* system needs genetic modification to prepare the NIV sensitivity, and wheat genome knowledge is still developing. Hence, the *Chlamydomonas reinhardtii* model system is a useful candidate for the evaluation of the cell system except for mammalian cells. 

*Chlamydomonas reinhardtii* is a monocellular green algae with chloroplasts, and it has been applied as an experimental material of photosynthesis, phototaxis or channelrhodopsin studies. Abundant genomic information has been gathered through these studies, and recently, the whole genome microarray of *C. reinhardtii* has been constructed [21]. Alexander *et al*. [22] evaluated trichothecene mycotoxins using the *C. reinhardtii* CC125 mt+ wild-type strain. It can grow under heterotrophic conditions with low light intensity and it is managed as a model plant organism in photosynthesis studies. McCormick [23] implied that this model is also useful for NIV evaluation and its genome analysis is making progress [24]. Taken together with those reports, the usefulness of *C. reinhardtii* for Toxicity evaluation is continuously increasing. In this study, the *C. reinhardtii* system was used for evaluating type B trichothecenes, and the appropriate testing conditions were examined for expanding information on the mycotoxin toxicities against plant cells. On the basis that *C. reinhardtii* is the model organism for photosynthesis study, the relationship between mycotoxins and lighting conditions was investigated. This study will provide information on phytotoxicity, which is useful for the protection and regulation of various agricultural environments including plant factories with Light-emitting diode (LED) lighting.

## 2. Results and Discussion

### 2.1. Mycotoxin Sensitivities to Trichothecenes and Lighting Conditions

**Figure 1:**
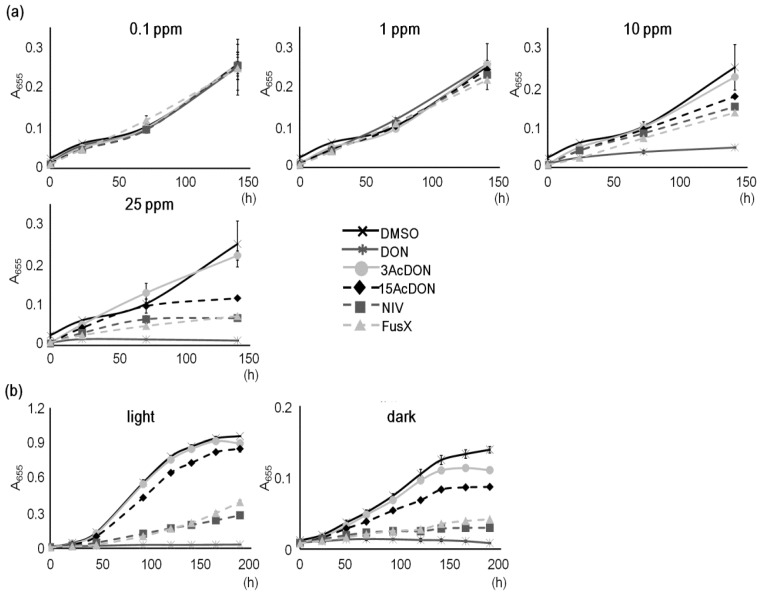
Evaluation conditions for type B trichothecenes in the *C. reinhardtii* testing system. (**a**) Influence of mycotoxin concentration on *C. reinhardtii* growth where cells were incubated in a multi-well plate with constant fluorescent light; (**b**) Influence of lighting conditions on *C. reinhardtii* growth where the light condition was 18 μmol m^−2^ s^−1^ of white Light-emitting diode (LED). Dimethyl sulfoxide (DMSO) was the control. A_655_ = absorbance 655 nm, Bars represent S.E. (*n* = 3). 0.1–25 ppm = 1.0 × 10^−4^–2.5 × 10^−2^ mg/mL.

*Chlamydomonas reinhardtii* has a heterotrophic character as it grows in nutrient-rich conditions although it is an autotroph as it photosynthesizes. By its photosynthesis ability, even the high-salt medium (HSM minimum medium; [25]), which lacks nutrients for heterotrophic growth, is able to sustain cell cultivation under abundant lighting and aeration. However, in a pre-cultivation test without aeration, the HSM medium slowed growth considerably (data not shown). The 96-well plate used in this study was not suitable for aeration treatments but was appropriate for the mycotoxin exposure test. Therefore, the TAP medium was prepared instead of the HSM medium as this enables heterotrophic growth, and a fluorescent ceiling light with a photosynthetic photon flux density (PPFD) of 6 μmol m^−^^2^ s^−^^1^ was applied to give some light for cell growth. The growth curves of *C. reinhardtii* with >1.0 × 10^−2^ mg/mL (10 ppm) mycotoxin conditions indicated changes of toxicities, and at 2.5 × 10^−2^ mg/mL (25 ppm), mycotoxins were readily identified (Figure 1a). Mycotoxin conditions of <10 ppm gave identical growth curves. The 3AcDON growth was similar to the control growth, but 15AcDON growth was less. The NIV and FusX growth curves indicated further low growth, and the DON growth indicated the lowest growth rate, which was derived from high toxicity. These results suggest that 25 ppm of mycotoxin is the best test condition for the evaluation of type B trichothecenes. Alexander *et al*. [22] found that 80 μM concentrations of mycotoxins was the best test condition and the 25 ppm of trichothecene used in this study is close to ~80 μM, thus agreeing with Alexander *et al*. despite using new trichothecene mycotoxins. NIV and FusX have not been previously compared using the *C. reinhardtii* testing system and NIV test condition studies that allow comparison with other trichothecenes have not been conducted. However, NIV has been reported to be the most toxic type B trichothecene compound [23]. This study also indicated that NIV is relatively toxic although DON had the most toxic character. These different results suggest that many parameters such as type of strain, static culture, media volume, lighting condition, and solvent (which are thought to be differential conditions) might have influenced the NIV toxicity result. 

In the barley leaf model study with fluorescent and incandescent lights, the lighting condition in the DON exposure test induced the bleaching of chloroplasts [15]. In this study, light and dark conditions were compared because the effect of complete dark with trichothecene mycotoxin was unknown and may induce differences in *C. reinhardtii* growth. Both conditions were prepared with the TAP medium, and the light condition was conducted at 18 μmol m^−2^ s^−1^ of white LED and the dark condition was conducted at 0 μmol m^−2^ s^−1^. The growth curves obtained from both conditions showed the same pattern although the maximal values in the dark condition remained low (Figure 1b). The toxicities of DON and 3AcDON are nearly identical, and those of NIV and FusX also closely resembled each other. In addition, 15AcDON toxicity was between 3AcDON and FusX and above all, DON almost totally inhibited growth. This means that type B trichothecenes did not induce the characteristic inhibitory effect on *C. reinhardtii* photosynthesis and growth with the TAP medium. This was not just due to the heterotrophic conditions provided, because cell growth with light was not that much better than in dark conditions. Teramoto *et al*. [26] has reported that the mRNA level of *Lhl4*, which encodes a distant relative of light-harvesting Chl-a/b proteins in *C. reinhardtii*, was elevated according to the increase of light intensity with the TAP medium. This indicates that photosynthesis was activated by the increase of light intensity. The difference between light and dark conditions was only a difference in growth rate, which agrees with this research without mycotoxin condition. Taken together, it suggests that the light conditions like white light (including fluorescent light and LED) do not influence the sensitivity of *C. reinhardtii* to trichothecene toxicity.

**Figure 2:**
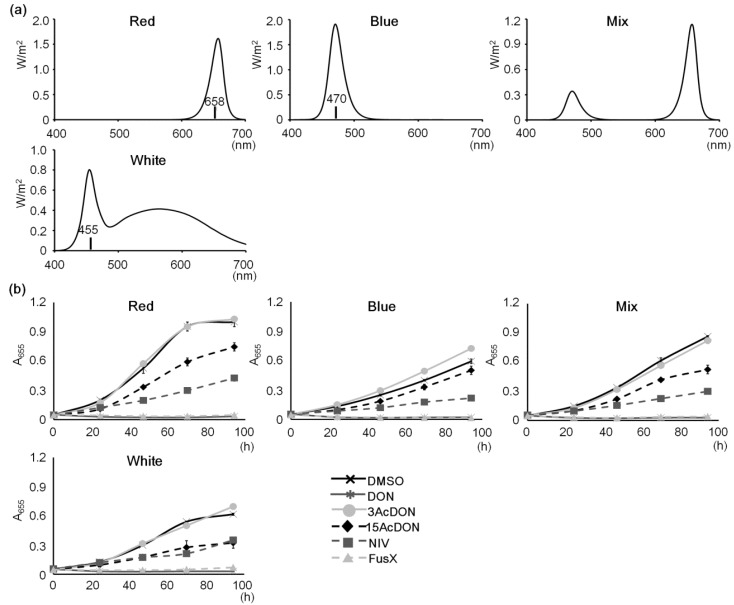
Spectral differences and sensitivities to mycotoxins. (**a**) LED spectral characters. Each graph is represented by the integration of irradiance. They only indicate spectral characters, not photosynthetic photon flux density (PPFD). Mix LED consists of the same components of red and blue LEDs. The maximal wavelength of each LED is indicated in the graph; (**b**) Growth test for comparing spectral characters. Lighting condition was 240 μmol m^−2^ s^−1^ of each LED. DMSO was the control. A_655_ = absorbance 655 nm, Bars represent S.E. (*n* = 3).

### 2.2. Differences of Spectra

Chlorophyll generally has two absorbance spectra; ~450 nm and ~660 nm. *Chlamydomonas reinhardtii* also has the same character of absorbance spectra. The blue spectrum, which is mainly 450 nm, is generally thought of as an important wavelength for *C. reinhardtii* growth because it regulates cell division and channel rhodopsin activation [27,28]. Additionally, it was reported that the blue spectrum induces more *Lhl4* mRNA accumulation than green, red, far-red, or dark conditions but not white light conditions [26]. Katsuda *et al*. [29] applied 18–36 μmol m^−2^ s^−1^ of blue LED in a photo-bioreactor to produce astaxanthin using a *Haematococcus pluvialis* system suggesting that blue LED is a useful testing condition. However, the most efficient growth of *C. reinhardtii* had been observed around the red spectra in a study that employed a chlorophyll synthesis mutant [30]. The red spectrum mainly consists of 660 nm and is also an important wavelength for photosynthesis. Differences in wavelength character influence various plant events as efficiencies of growth or photosynthesis. Various commercial LEDs can be obtained easily including many that carry appropriate bands for photosynthesis. Hence, in this study, blue, red, mix, and white LED conditions were tested (Figure 2a). A mix was prepared with a red-to-blue ratio of three to one. Red and blue LEDs were a bullet type, whereas the white LED, used as a general lighting control, was a bulb type. Each PPFD was set to 240 μmol m^−2^ s^−1^. All the growth data indicated the same trend to mycotoxin toxicities (Figure 2b). The 3AcDON condition did not indicate a characteristic difference compared with the control. Meanwhile, 15AcDON and DON indicated moderate or heavy toxicities, respectively. NIV indicated moderate changes, and FusX indicated high toxicities of the same degree as DON. Except under white LED conditions, NIV exposure indicated higher toxicities than that of 15AcDON. The most characteristic difference was in cell growth rates between LED spectra conditions. The red LED condition was able to obtain the most characteristic differences because it was the best growth condition in this study. This growth trend corresponds with another study that found maximal chlorophyll content and cell volume in the red spectrum [30]. The result of this study suggests that red spectrum lighting is the most efficient evaluation condition because it gives rapid growth and characteristic growth rates. However, in blue and white LED conditions (both with a low contribution of red spectra), it seems that the toxicity level of 15AcDON was changing. Therefore, spectrum regulation for trichothecene mycotoxin evaluation should be employed to understand the relationship between mycotoxin toxicity and photosynthesis. The sun spectrum is constantly changing, and recent studies suggest that fungi have light-regulation systems [31,32]. Taken together, it is thought that this is a good approach to obtain fundamental information about the relationship between fungal invasion mechanisms and the day-length responses of plants.

### 2.3. Photon Flux Conditions Affect FusX Sensitivity

**Figure 3:**
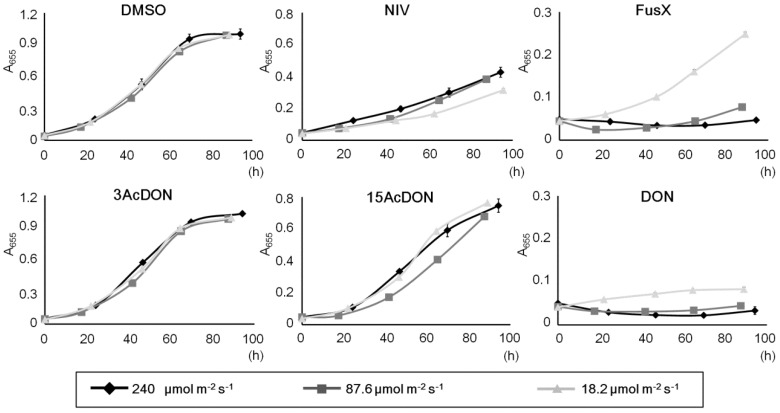
Relationship between trichothecenes and photon flux. Photon flux density (PFD) of red LED is regulated at 18.2, 87.6, and 240 μmol m^−2^ s^−1^. DMSO was the control. A_655_ = absorbance 655 nm, Bars represent S.E. (*n* = 3).

In this *C. reinhardtii* experimental system, a sufficient dose of PPF conditions enabled efficient growth and the identification of clear changes (Figure 2b). However, compared with lower dose conditions like in Figure 1, FusX did not grow as well as DON. It was not clear whether this problem was an accidental event, and therefore the influence of changes of PPFD was investigated. The red LED condition was selected because it results in the most efficient growth, and PPFDs were set up from 18.2 to 240 μmol m^−2^ s^−1^. The low PPFD was almost identical to the lighting condition of Figure 1b, the high PPFD value was almost identical to the lighting condition of Figure 2b, and the middle value was set to 87.6 μmol m^−2^ s^−1^. Except for FusX, almost all conditions did not give a significant change in growth curve (Figure 3). A low PPFD condition gave the most efficient growth in FusX and the middle and high PPFD conditions indicated extremely weak growth. Only 18.2 μmol m^−2^ s^−1^ light condition indicated a statistical significance (*P* < 0.05). These results corresponded to the trends in Figure 1 and Figure 2. Control conditions and 3AcDON conditions did not have any changes in growth curves. These results suggest that a change in PPFD only influences the sensitivity of *C. reinhardtii* to FusX. A higher toxicity of FusX than NIV has been reported from various cellular test conditions [10,33]. However, the difference in the plant pathogen toxicity mechanism between NIV and FusX is not clear. The lighting regulation will help to address this problem.

## 3. Experimental Section

### 3.1. Strain, Medium, and Mycotoxins

A colony of *C. reinhardtii* wild-type strain 137C was picked from slant culture on Tris-Acetate-Phosphate (TAP) medium agar [34]. The TAP medium consists of three stock solutions. The TAP-salts solution was prepared from 40 g of NH_4_Cl, 10 g of MgSO_4_.7H_2_O, 5 g of CaCl_2_.2H_2_O, 242 g of Tris base, and 100 mL of acetic acid per 1 L. The phosphate solution was prepared from 10.8 g of K_2_HPO_4_ and 5.6 g of KH_2_PO_4_. The trace elements solution was prepared from 5 g of Na_2_.EDTA, 2.2 g of ZnSO_4_.7H_2_O, 1.14 g of H_3_BO_3_, 0.51 g of MnCl_2_.4H_2_O, 0.16 g of CoCl_2_.6H_2_O, 0.16 g of CuSO_4_.5H_2_O, 0.11 g of (NH_4_)6Mo_7_O_24_.4H_2_O, 0.5 g of FeSO_4_.7H_2_O, and 1.6 g of KOH per 1 L. *Chlamydomonas reinhardtii* was pre-incubated at 100 rpm rotation, at 25 °C, and constant lighting for more than 3 days. Each mycotoxin, DON, 3-acetyl-deoxynivalenol (3AcDON), 15-acetyl-deoxynivalenol (15AcDON), NIV, and fusaronon-X (FusX, 4acetyl-NIV) was dissolved in dimethyl sulfoxide (DMSO) to prepare a stock solution of 2000 ppm. A solution of only DMSO was used as a control.

### 3.2. Light Source

Light-emitting diode (LED) conditions were manually constructed on an LED platform (SPL-100-CC; REVOX, Kanagawa, Japan) with red (660 nm) and blue (470 nm) diodes, and photon flux density (PFD) was modulated by a pulse-width modulation dimmer controller. Mix LED consisted of both red and blue LEDs with a three-to-one ratio. Meanwhile, a white bulb-shaped LED was used as a general light control. Each spectrum of LED irradiation was measured by an illuminance spectrophotometer (CL-500A; Konica Minolta, Tokyo, Japan) as irradiances (W/m^2^). Total irradiances of spectra from 400 to 700 nm were counted and the photosynthetic photon flux density (PPFD) of each spectrum condition was calculated with the following formula:

[PPFD (μmol m^−2^ s^−1^) = ([irradiance (W/m^2^) × spectrum (m) × 10^−9^]/[Planck’s constant (6.626 × 10^−34^; J.s) × Speed of light (2.998 × 10^8^; m/s) × Avogadro constant (6.022 × 10^23^; mol^−1^)]) × 10^6^]

### 3.3. Growth Test Condition

TAP-based cell cultures were incubated with 1.0 × 10^−^^4^–2.5 × 10^−^^2^ mg/mL (0.1–25 ppm) of the mycotoxins, or DMSO only as a control. Each total culture volume was adjusted to 200 μL, and DMSO, which is a solvent, was adjusted to the low concentration value because an increase in DMSO influences *C. reinhardtii* growth negatively [35]. Sorted cultures in 96-well flat bottom micro plates were placed under the LED lighting at 25 °C. Culture plates were transferred into a plate reader (iMark; Bio-Rad, Hercules, CA, USA) once a day, and then absorbance at 655 nm was measured. Each test was conducted with triplet samples. Growth curves of the FusX exposure test were analyzed using the T-test, and 240 μmol m^−2^ s^−1^ lighting condition was defined as a control.

## 4. Conclusions

In this study, trichothecene evaluation conditions were determined using *C. reinhardtii* as a model system. This system is advantageous for the evaluation of NIV toxicity where yeast or higher plants cannot be used as a test system. When evaluating mycotoxin toxicities, the effects of lighting conditions have not really been investigated. The results of this study suggested that differences in the light spectrum or incident density influence several trichothecenes. Recently, the whole genome microarray of *C. reinhardtii* has been constructed [21], and therefore the *C. reinhardtii* system has the ability to bring gene expression data derived from the differences of sensitivity against type B trichothecenes. Because the finding derived from gene expression analysis can be integrated with various cell systems, this system will become more useful, especially for investigating the relationship between the photosynthetic pathway and mycotoxins. 
